# Nanopore sequencing reveals a diversity of microorganisms in ticks from Ethiopia

**DOI:** 10.1007/s00436-025-08520-1

**Published:** 2025-06-30

**Authors:** Electra F. Chadd, Koray Ergunay, Bersissa Kumsa, Brian P. Bourke, Ben S. Broomfield, Lewis S. Long, Yvonne-Marie Linton

**Affiliations:** 1 Uniformed Services University of the Health Sciences, 4301 Jones Bridge Road, Bethesda, MD 20814 USA; 2 Department of Entomology, Smithsonian Institution National Museum of Natural History (NMNH), Washington, DC 20560 USA; 3 Department of Parasitology, College of Veterinary Medicine and Agriculture, Addis Ababa University, PO BOX 34, Bishoftu, Ethiopia; 4 University of Manchester, Manchester, M13 9PL UK

**Keywords:** Ethiopia, Ixodidae, Metagenomics, Nanopore sequencing, Zoonosis

## Abstract

Ethiopia is home to a diverse group of ixodid ticks that are known to transmit disease to both animals and humans. Recent advances in metagenome sequencing show there is more microorganism diversity found in ticks than previously known. Compared to amplicon-based gene identification methods, agnostic metagenomic sequencing provides broader insights into the diversity of microorganisms in ticks, providing knowledge that will better enable public health measures in preventing the spread of zoonotic disease. In the present study, metagenomic sequencing was used to look at the microbial diversity of ticks collected from livestock in Ethiopia. This study identified six bacterial genera (*Coxiella*, *Francisella*, spotted-fever group (SFG) *Rickettsia*, *Spiroplasma*, *Ehrlichia*, and *Borrelia*), one genus of eukaryotic parasite (*Babesia* sp.), and one virus species (*Parapoxvirus bovinestomatitis*) from 154 tick pools representing 22 species of ticks among four genera (*Amblyomma*, *Haemaphysalis*, *Hyalomma*, and *Rhipicephalus*). We were able to differentiate between pathogenic and nonpathogenic microorganisms, highlighting concerns among traditional gene-targeted screening methods. Among all pooled samples, the predominant microorganisms included *Coxiella-*like endosymbionts (55.2%), SFG *Rickettsia* (38.3%), and nonpathogenic *Francisella* spp. (26.0%). *Rickettsia africae* was the predominant pathogenic agent detected, and phylogenetic analysis of two samples from *A. gemma* and *A. variegatum* confirmed the presence of *R. africae.* This study highlights the power of metagenomics applied to potential vectors of zoonotic disease, and it expands the knowledge on tick-pathogen associations in Ethiopia.

**Supplementary Information:**

The online version contains supplementary material available at 10.1007/s00436-025-08520-1.

## Introduction

Vector-borne pathogens present a significant public health threat to humans and food security, and ticks are commonly cited as being second only to mosquitoes in their vectorial capacity for pathogens (Parola and Raoult 2001; de la Fuente et al. 2008). Because ticks parasitize vertebrates in almost every region of the world, they are able to circulate a wide range of pathogenic agents (Parola and Raoult 2001; Estrada-Peña et al. 2017). The extent of zoonotic spillover of tick-borne pathogens from enzootic cycles remains largely unknown due to challenges in tick identification and the recognition of tick-borne disease etiology (Dantas-Torres et al. 2012; Paguem et al. 2023). Surveillance of tick-borne pathogens and microbial agents mostly depends on pathogen-specific pathogen assays that target a priori gene regions of known agents. Researchers have harnessed recent advances in sequencing technology and adapted high-throughput sequencing platforms to investigate a wider range of pathogens and microbial communities of arthropods (Mohamed et al. 2022). Among these emerging molecular approaches is agnostic metagenomic sequencing, which does not rely on the detection of target genes and constitutes an alternative approach to microbial surveillance that uncovers a wider spectrum of microbial diversity that ticks may harbor (Ergunay et al. 2022). This approach has the capacity to simultaneously detect multiple known and unknown agents and provide sequence data suitable for phylogenetic analysis (Zhu et al. 2020; Kipp et al. 2023).

Considering their close association to humans, the role ticks and domesticated hosts play in the cycle of zoonotic disease makes ticks a medically important group of arthropods worldwide (Wikel 2018; Rochlin and Toledo 2020). This relationship can be seen in areas such as Ethiopia, where pastoralism is a major livelihood. The live animal trade in Ethiopia accounts for 90% of the country’s national export, and considerable human and livestock migration occurs through Ethiopia and the Horn of Africa to countries across the African continent and into the Arabian Peninsula (Abduletif 2019; Zekarias 2023). This large and moving livestock population puts humans at the interface of zoonotic spillover (Zinsstag et al. 2020; CSA 2022; FAO 2024). Tick and pathogen distribution patterns are changing for reasons such as population migration, urbanization, and climate change (Estrada-Pena 2008; Margouras et al. 2020). The resultant changes affect host, vector, and pathogen dynamics, and they can have potentially negative implications on livestock production systems as well as pastoralist livelihood (Gharbi et al. 2020).

Records of tick-borne bacteria, parasites, and viruses have been reported from an extensive literature review of ticks and tick-borne pathogens found in Ethiopia (Lilak and Pecor et al. 2024). However, metagenomic analysis has not yet been applied to any studies to date, leaving open the potential for additional tick-borne microorganisms to be discovered. Moreover, opportunistic and symbiotic microorganisms distinguished by particular gene regions may be bi-laterally misidentified as their closely related pathogenic organisms or vice versa, leading to an over- or under-representation of true pathogen prevalence and associated risk to humans or animals (Öhrman et al. 2021). Our team has reported nonpathogenic tick-associated microorganism findings via agnostic sequencing methods in Kenya (Ergunay et al. 2022), and similar findings have been reported in Tunisia (Benyedem et al. 2022), but no such records yet exist for Ethiopia. This study aims to holistically characterize the tick-borne microorganism associations in ticks from Ethiopia using metagenomic approaches for the first time.

## Materials and methods

### Study area and design

**Fig. 1:**
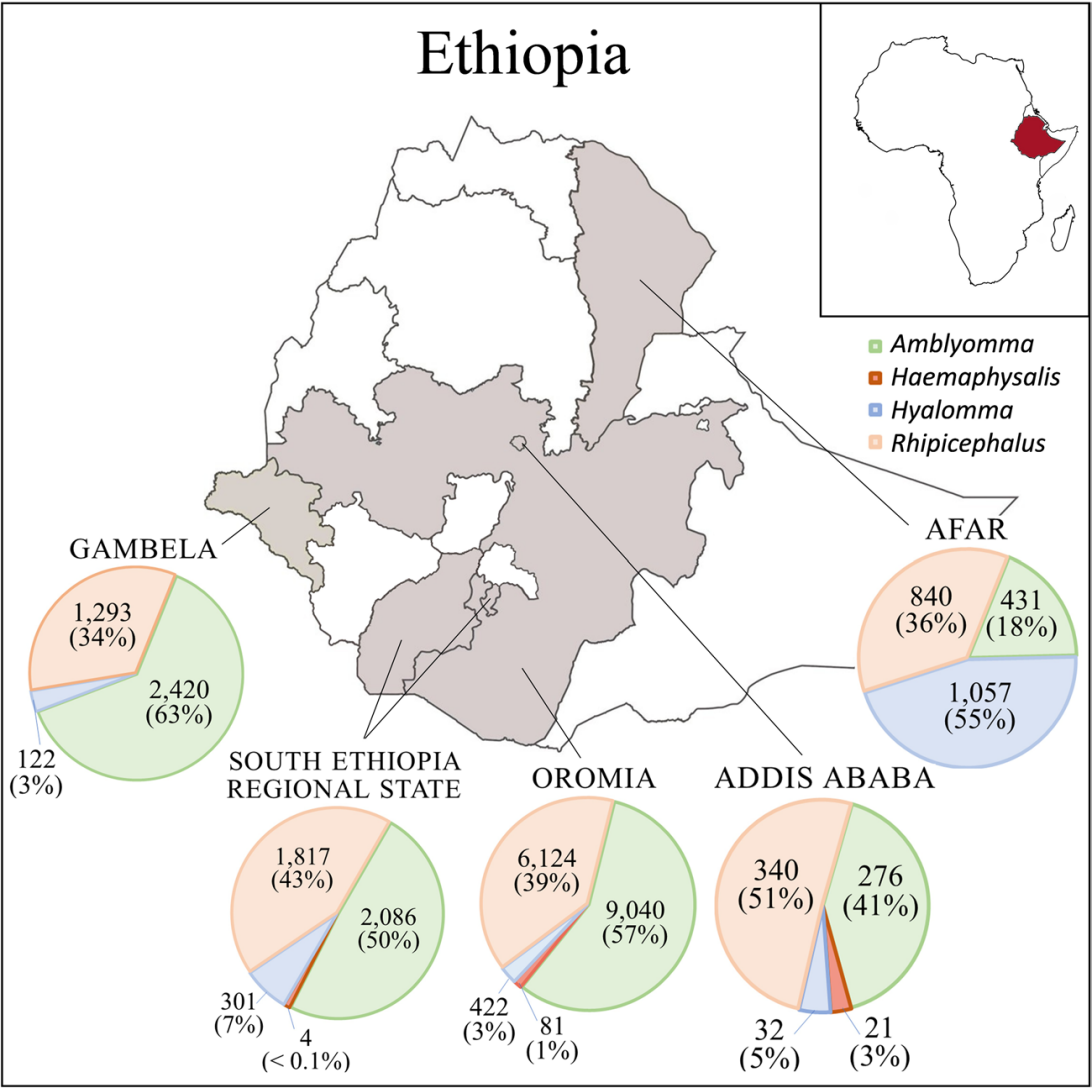
Map of study area and distribution of ticks [*n*, (%)] by genus collected from livestock across five geopolitical divisions in Ethiopia. In Addis Ababa, we found 12 tick species (*A. cohaerens*, *A. gemma*, *A. variegatum*, *Hae. aciculifer*, *Hae. leachi*, *Hae. spinulosa* subgroup, *Hy. rufipes*, *Hy. truncatum*, *Rh. decoloratus*, *Rh. evertsi*, *Rh. pulchellus*, and *Rh. sanguineus* s.l.). In Afar, we found ten tick species (*A. gemma*, *A. lepidum*, *A. variegatum*, *Hy. dromedarii*, *Hy. excavatum*, *Hy. rufipes*, *Hy. truncatum*, *Rh. evertsi*, *Rh. praetextatus*, and *Rh. pulchellus*). In Gambela, we found seven tick species (*A. lepidum*, *A. variegatum*, *Hy. rufipes*, *Rh. annulatus*, *Rh. decoloratus*, *Rh. evertsi*, and *Rh. praetextatus*). We found 16 tick species in both Oromia (*A. cohaerens*, *A. gemma*, *A. lepidum A. variegatum*, *Hae. aciculifer*, *Hae. leachi*, *Hy. rufipes*, *Hy. truncatum*, *Rh. bergeoni*, *Rh. decoloratus*, *Rh. evertsi*, *Rh. lunulatus*, *Rh. praetextatus*, *Rh. pravus*, *Rh. pulchellus*, and *Rh. sanguineus* s.l.) and South Ethiopia Regional State (*A. cohaerens*, *A. gemma*, *A. lepidum A. variegatum*, *Hae. aciculifer*, *Hae. parmata*, *Hy. impeltatum*, *Hy. rufipes*, *Hy. truncatum*, *Rh. bergeoni*, *Rh. decoloratus*, *Rh. evertsi*, *Rh. lunulatus*, *Rh. praetextatus*, *Rh. pravus*, and *Rh. pulchellus*)

We employed a cross-sectional study that looked at tick-associated microorganisms in ixodid ticks collected from Ethiopia (Fig. 1). During veterinary visits in 2021 and 2022, ticks were found and manually collected after full-body examination of livestock (e.g., camel, cattle, horse, goat, and sheep) and other domestic animals. All ticks found were removed using forceps and immediately stored in plastic vials containing 75% ethanol. Information on sampling location and animal host was recorded. Ticks were transported back to the parasitology laboratory at Addis Ababa University College of Veterinary Medicine for identification.

### Tick identification and nucleic acid extraction

All ticks (male and female adults, and nymphs) were morphologically identified using a stereomicroscope and available taxonomic keys (Hoogstraal 1956; Morel and Balis 1976; Morel 1980; Pegram et al. 1981; Pegram et al. 1987a, b; Pegram 1989; Walker et al. 2000; Walker et al. 2014; Apanaskevich and Horak 2008a, b; Apanaskevich et al. 2008; Apanaskevich and Horak 2009). Identified, ethanol-stored ticks were shipped to the Smithsonian Institution Museum Support Center (Suitland, MD, USA) for pathogen screening. Sub-samples of ticks were chosen for comprehensive coverage of tick species and sex. Although the tick species surveyed in this study may occasionally feed on humans, additional tick pools were tested for the predominant species known to feed on humans (*A. variegatum*, *Hy. rufipes*, *Hy. truncatum*, and *Rh. evertsi*) (Guglielmone and Robbins 2018). Phenol–chloroform nucleic acid extractions were conducted on individual ticks from a total of five geopolitical regions and spanning 22 species and four genera using the AutoGenprep 965 platform (AutoGen Inc., Holliston, MA) and the associated extraction kit following the manufacturer’s instructions. Only DNA microorganisms were targeted, as all samples were stored in ethanol. Following individual extractions, pools of 1–5 ticks were created using a subsample of the individual extracts, by site, host, tick species, and sex. In all, 154 pools representing 22 unique species among four genera (*Amblyomma*, *Haemaphysalis*, *Hyalomma*, and *Rhipicephalus*) were prepared for nanopore sequencing.

### Nanopore sequencing

Pooled samples underwent cDNA library synthesis, purification, and quantification following that of Nelson et al. (2024), using the Remote Emerging Disease Intelligence-NETwork (REDI-NET) T-4 Tick Testing protocol (10.17504/protocols.io.kxygx3ozzg8j/v2). Nucleic acids were repaired and prepared for labeling with the NEBNext Ultra II End Repair/dA-Tailing Module and the NEBNext FFPE DNA Repair Module (New England Biolabs). The Native Barcoding Kit 96 V14, SQK-NBD114.96 (Oxford Nanopore Technologies (ONT), Oxford, UK) was used with NEB Blunt/TA Ligase Master Mix (New England Biolabs®, Ipswich, MA) for barcode ligation. Pools of 16 barcoded samples (each from prepared nucleic acid pools) were made and purified with Agencourt AMPure XP beads (Beckman Coulter Biosciences, Indianapolis, USA). Adapters from the Native Barcoding Kit 96 V14, SQK-NBD114.96 (ONT) were ligated with NEBNext Quick Ligation Module (New England Biolabs). The prepared libraries were quantified with Qubit 1X dsDNA HS Assay Kit (Thermo Fischer Scientific, Waltham, MA). A total volume of 12 µL of reach library was loaded onto R10.4.1 flow cells (ONT) and run on an ONT GridION X5 sequencer for 48 h.

### Data analysis

Raw reads were trimmed to remove barcode labels using Porechop (https://github.com/bonsai-team/Porechop_ABI) (Bonenfant et al. 2023) and filtered using nanofilt (https://github.com/wdecoster/nanofilt) to remove short (≤ 100 base pairs) and low quality (*Q* < 9) reads. Tick host genomes were then removed from the data using representatives from all available *Ixodidae* genera in the NCBI Reference Genome library (*Hy. asiaticum*, GCA_013339685; *Hae. longicornis*, GCA_013339765; *A. americanum*, GCA_030143305; *Rh. sanguineus*, GCF_013339695; *Ixodes scapularis*, GCF_016920785; *Dermacentor andersoni*, GCF_023375885) and the tools Minimap2 v2.24 (Li 2018) and Samtools v1.9 (Danecek et al. 2021). The data was then aligned to the National Center for Biotechnology Information (NCBI) nonredundant (NR) database using DIAMOND v2.0.14 (Buchfink et al. 2021) and visualized using MEGAN6 (v6.23.2) (Huson et al. 2016). Reads classified as nodes of taxonomic interest were extracted as FASTA files and assembled using the Geneious Prime v2024.0.7 (Biomatters Ltd., Auckland, New Zealand) de novo assembly tool. Sequences were grouped by similarity according to taxonomic family and extracted into FASTA files. Consensus sequences were then mapped to a curated set of reference genomes (Supplementary file 1), and phylogenetic trees were constructed using MEGA X v10.2.6 (Kumar et al. 2018).

## Results

In all, we collected 26,218 total ticks from eight different animals [camel (8.7%), cattle (78.3%), dog (0.8%), goat (1.4%), horse (1.9%), sheep (8.8%), cat (0.01%), and donkey (0.1%)] across five geopolitical regions in Ethiopia [Addis Ababa (2.5%), Afar (8.7%), Gambela (14.3%), Oromia (58.6%), and South Ethiopia Regional State (15.8%)]. Ticks were morphologically identified to 22 unique species belonging to four genera [*Amblyomma* (53.3%), *Haemaphysalis* (0.4%), *Hyalomma* (7.2%), and *Rhipicephalus* (39.0%)] (Table 1). Of the ticks collected, 154 pooled samples (1–5 ticks per pool) were surveyed for microorganisms using nanopore sequencing, and 128 (83.1%) were found to be positive for at least one of 13 unique microorganisms across all sampled locations (Tables 1 and 2). Among the microorganisms detected, we found six bacterial genera (*Coxiella*, *Francisella*, *Rickettsia*, *Spiroplasma*, *Ehrlichia*, and *Borrelia*), one eukaryotic parasite genus (*Babesia*), and one double-stranded DNA virus (*Parapoxvirus bovinestomatitis* [bovine papular stomatitis virus (BPSV)]). Opportunistic and symbiotic bacteria (*Coxiella-*like endosymbionts, *F. opportunistica*, *F. persica*, *Spiroplasma* endosymbionts, and *Borrelia* sp.) were found among agents known to be pathogenic (SFG *Rickettsia*, *C. burnetti*, *E. canis*, *E. ruminatium*, *F. tularensis* subsp. *novicida*, *S. ixodetis*, and BPSV). *Coxiella-*like endosymbionts were the most prevalent microorganisms found (53.9%). Among pathogenic microorganisms, SFG *Rickettsia* species were most prevalent, with *R. africae* being the predominant species detected in 14.9% of the pools.

**Table 1:** Tick species and microorganisms found by geopolitical region found across Ethiopia. (*A*. = *Amblyomma*, *Hae*. = *Haemaphysalis*, *Hy*. = *Hyalomma*, *Rh*. = *Rhipicephalus*)

	Geopolitical region				
	Addis Ababa	Afar	Gambela	Oromia	South Ethiopia Regional State
Tick species					
*A. cohaerens*	X			X	X
*A. gemma*	X	X		X	X
*A. lepidum*		X	X	X	X
*A. variegatum*	X	X	X	X	X
*Hae. aciculifer*	X			X	X
*Hae. leachi*	X			X	
*Hae. parmata*					X
*Hae. spinulosa* subgroup	X				
*Hy. dromedarii*		X			
*Hy. excavatum*		X			
*Hy. impeltatum*					X
*Hy. rufipes*	X	X	X	X	X
*Hy. truncatum*	X	X		X	X
*Rh. annulatus*			X		
*Rh. bergeoni*				X	X
*Rh. decoloratus*	X		X	X	X
*Rh. evertsi*	X	X	X	X	X
*Rh. lunulatus*				X	X
*Rh. praetextatus*		X	X	X	X
*Rh. pravus*				X	X
*Rh. pulchellus*	X	X		X	X
*Rh. sanguineus* s.l	X			X	
Microorganism					
*Coxiella*-like endosymbionts	X	X	X	X	X
*C. burnetii*			X		
Opportunistic *Francisella*	X	X	X	X	X
*F. tularensis novicida*	X	X		X	X
SFG *Rickettsia*	X	X	X	X	X
*R. africae*	X	X	X	X	X
Opportunistic *Spiroplasma*	X		X	X	X
*S. ixodetis*	X				
*E. canis*		X			X
*E. ruminatium*				X	X
*Borrelia* sp.	X			X	
*Babesia* sp.		X			
BPSV			X

**Table 2:** Microorganism detection prevalences in pooled ticks according to tick genus. *n* (%) (SFG = Spotted fever group; BPSV = bovine papular stomatitis virus)

	Bacteria						Virus	Parasite
Tick genus (*n* total pools)	*Coxiella*	*Francisella*	SFG* Rickettsia*	*Spiroplasma*	*Ehrlichia*	*Borrelia*	BPSV	*Babesia*
*Amblyomma* (43)	27 (62.8%)	-	23 (53.4%)	-	2 (4.7%)	-	-	-
*Haemaphysalis* (6)	5 (83.3%)	1 (16.7%)	3 (60.0%)	-	-	1 (16.7%)	-	-
*Hyalomma* (46)	7 (13.0%)	29 (63.0%)	18 (39.1%)	2 (4.4%)	3 (6.5%)	-	-	1 (2.2%)
*Rhipicephalus* (59)	46 (78.0%)	10 (16.9%)	15 (25.4%)	4 (6.8%)	1 (1.7%)	1 (1.7%)	2 (3.9%)	-
Total (154)	85 (55.2%)	40 (26.0%)	59 (38.3%)	6 (3.9%)	6 (3.9%)	2 (1.3%)	2 (1.3%)	1 (0.06%)

### Coxiella

Bacteria from the genus *Coxiella* were the most common microorganism encountered, with *Coxiella-*like endosymbionts, accounting for 97.6% (83/85) of all *Coxiella* detected (Table 3). Only one pool each of *Rh. annulatus* and *Rh. evertsi* were positive for *C. burnetii*, the causative agent of Q fever. With the exception of one *Hae. parmata* tick pool that did not yield any microorganisms, all pools belonging to the genus *Haemaphysalis* were positive for *Coxiella-*like endosymbionts. Pools of *Rhipicephalus* and *Amblyomma* also showed high prevalence of *Coxiella-*like endosymbionts (74.6% and 62.8%, respectively). *Coxiella-*like endosymbionts were detected in all *Rhipicephalus* species, with *Rh. pulchellus* (*n* = 4) and *Rh. sanguineus* s.l. (*n* = 1) pools exhibiting 100% infection. All pools of *A. gemma* (*n* = 3) tested positive for *Coxiella*-like endosymbionts. *Hyalomma* pools showed lower prevalence of *Coxiella*-like endosymbionts (13.0%), with 66.7% of *Hy. dromedarii* (*n* = 3), 14.3% of *Hy. rufipes* (*n* = 21), and 11.1% of *Hy. truncatum* (*n* = 18) testing positive (Table 3).

**Table 3:** Tick pools positive for *Coxiella* spp. *n* (%) (*A*. = *Amblyomma*, *Hae*. = *Haemaphysalis*, *Hy*. = *Hyalomma*, *Rh*. = *Rhipicephalus*)

Tick species (*n*)	*Coxiella*-like endosymbionts	*C. burnetii*
*A. cohaerens* (8)	6 (75.0%)	-
*A. gemma* (3)	3 (100%)	-
*A. lepidum* (3)	2 (66.7%)	-
*A. variegatum* (29)	16 (55.2%)	-
*Hae. aciculifer* (3)	3 (100%)	-
*Hae. leachi* (2)	2 (100%)	-
*Hy. dromedarii* (3)	2 (66.7%)	-
*Hy. rufipes* (21)	3 (14.3%)	-
*Hy. truncatum* (18)	2 (11.1%)	-
*Rh. annulatus* (5)	3 (60.0%)	1 (20.0%)
*Rh. bergeoni* (3)	2 (66.7%)	-
*Rh. decoloratus* (13)	10 (76.9%)	-
*Rh. evertsi* (18)	14 (77.8%)	1 (5.6%)
*Rh. lunulatus* (3)	2 (66.7%)	-
*Rh. praetextatus* (8)	6 (75.0%)	-
*Rh. pravus* (4)	2 (50.0%)	-
*Rh. pulchellus* (4)	4 (100%)	-
*Rh. sanguineus* (1)	1 (100%)	-
	83 (53.9%)*	2 (1.3%)*

*Overall prevalences were calculated from all tick pools tested (*n* = 154)

### Francisella

*Francisella* spp. were most prevalent in *Hyalomma* pools (63.0%), followed by *Rhipicephalus* (16.9%), then *Haemaphysalis* (16.7%) (Table 4). While all *Amblyomma* pools tested negative for *Francisella* microorganisms, three tick genera (*Haemaphysalis*, *Hyalomma*, and *Rhipicephalus*) harbored opportunistic *Francisella* (*F. persica* and *F. opportunistica*), with all *Hyalomma* pools testing positive. Furthermore, *Hy. impeltatum* (33.3%), *Hy. rufipes* (27.8%), and *Hy. truncatum* (5.6%) had pools that yielded sequences mapping to *F. tularensis novicida*, a pathogenic variant of *F. tularensis.*

**Table 4:** Tick pools positive for *Francisella* spp. *n* (%) (*Hae*. = *Haemaphysalis*, *Hy.* = *Hyalomma*, *Rh.* = *Rhipicephalus*)

Tick species (*n*)	Opportunistic *Francisella* spp.	*F. tularensis novicida*
*Hae. leachi* (2)	1 (50.0%)	-
*Hy. dromedarii* (3)	2 (66.7%)	-
*Hy. excavatum* (1)	1 (100%)	-
*Hy. impeltatum* (3)	2 (66.7%)	1 (33.3%)
*Hy. rufipes* (21)	12 (57.1%)	5 (27.8%)
*Hy. truncatum* (18)	12 (66.7%)	1 (5.6%)
*Rh. annulatus* (5)	1 (20.0%)	-
*Rh. evertsi* (18)	1 (5.6%)	-
*Rh. praetextatus* (8)	1 (12.5%)	-
	33 (21.4%)*	7 (4.5%)*

*Overall prevalences were calculated from all tick pools tested (*n* = 154)

### Spotted-fever group *Rickettsia*

SFG *Rickettsia* were detected in all genera tested, with the highest prevalence in *Haemaphysalis* (60.0%), followed by *Amblyomma* (53.4%), *Hyalomma* (39.1%), and *Rhipicephalus* (38.3%). All pools of *Rh. lunulatus* (*n* = 3), 50% of *Hae. leachi* (*n* = 2), and *Rh. praetextatus* (*n* = 8) pools were positive for SFG *Rickettsia*. The predominant SFG *Rickettsia* found was *R. africae*, and all species of *Amblyomma* had at least one pool where *R. africae* was detected: *A. variegatum* (37.9%), *A. gemma* (33.3%), *A. lepidum* (33.3%), and *A. cohaerens* (25.0%) (Table 5).

**Table 5:** Tick pools positive for *Rickettsia* spp*. n* (%) (*A*. = *Amblyomma*, *Hae*. = *Haemaphysalis*, *Hy*. = *Hyalomma*, *Rh*. = *Rhipicephalus*)

Tick species (*n*)	*R. africae*	SFG *Rickettsia* (other)
*A. cohaerens* (8)	2 (25.0%)	2 (25.0%)
*A. gemma* (3)	1 (33.3%)	1 (33.3%)
*A. lepidum* (3)	1 (33.3%)	-
*A. variegatum* (29)	11 (37.9%)	5 (17.2%)
*Hae. aciculifer* (3)	-	2 (33.3%)
*Hae. leachi* (2)	-	1 (50.0%)
*Hy. impeltatum* (3)	-	1 (33.3%)
*Hy. rufipes* (21)	3 (14.3%)	12 (57.1%)
*Hy. truncatum* (18)	1 (5.6%)	1 (5.6%)
*Rh. annulatus* (5)		1 (20.0%)
*Rh. bergeoni* (3)	1 (33.3%)	1 (33.3%)
*Rh. evertsi* (18)	1 (5.6%)	1 (5.6%)
*Rh. lunulatus* (3)	-	3 (100%)
*Rh. praetextatus* (8)	2 (25.0%)	4 (50.0%)
*Rh. pravus* (4)	-	1 (25.0%)
	23 (14.9%)*	36 (23.4%)*

*Overall prevalences were calculated from all tick pools tested (*n* = 154)

**Fig. 2:**
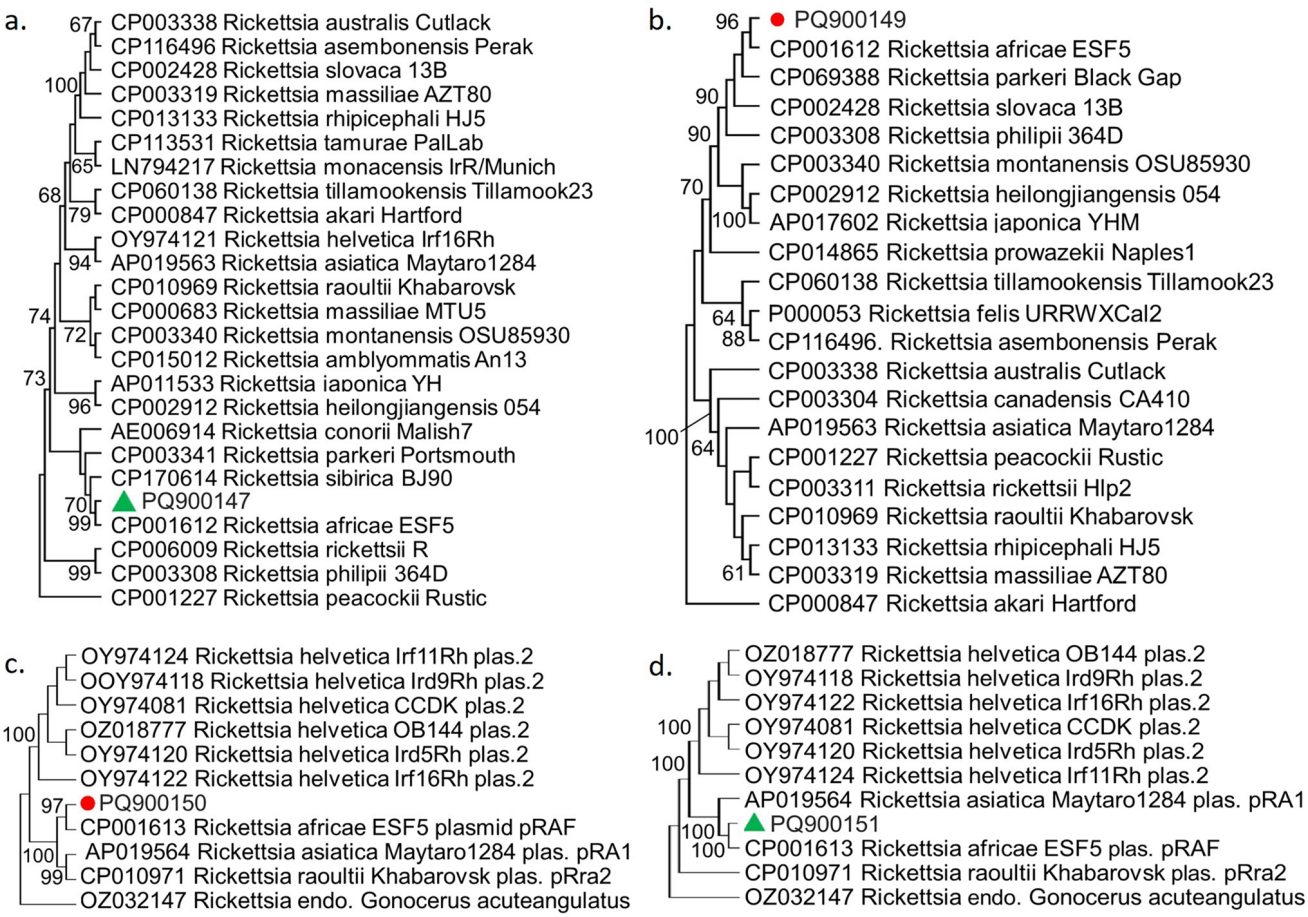
The maximum likelihood consensus trees of sequences mapping to *Rickettsia africae*. **a** bcr2 (PQ900147, 1124 nucleotides), **b** ppnK/hydrolase (PQ900149, 174 nucleotides), **c** plasmid (PQ900150, 805 nucleotides), and **d** plasmid (PQ900151, 786 nucleotides). The trees are generated for 500 replicates with the Tamura 3-parameter model with invariant sites, and bootstrap values lower than 60 are not shown. *Rickettsia* strains are indicated by GenBank accession number, name, and isolate identifier

Through phylogenetic analysis of chromosomal and plasmid-based contigs, the infecting SFG *Rickettsia* spp. was determined to be *R. africae* in two pooled samples (one each from *A. gemma* and *A. variegatum*). The chromosomal contigs covered bcr2 and ppnK, encoding for MFS-type bicyclomycin resistance protein and putative inorganic polyphosphate/ATP-NAD kinase, respectively. Individual maximum likelihood analyses placed these and plasmid sequences in separate clusters among *R. africae* (Fig. 2).

### Other microorganisms

Other agents identified in this study include species of *Ehrlichia*, *Spiroplasma*, and *Borrelia* (Table 6). *E. ruminantium* was detected in a pool of *Hyalomma dromedarii* (33.3%) and in two pools of *H. impeltatum* (66.7%), while *E. canis* was found in two pools of *A. cohaerens* (25.0%). *Spiroplasma* endosymbionts were present in four pools of *Rh. decoloratus* (30.7%), as well as in pools of *H. truncatum* (5.6%) and *H. rufipes* (4.8%). *Spiroplasma ixodetis* was identified in *R. decoloratus* (7.7%). *Borrelia* spp. were detected in *Hae. aciculifer* (33.3%) and *Rhipicephalus evertsi* (5.6%). BPSV, a double-stranded DNA virus responsible for bovine papular stomatitis, was found in ethanol-preserved samples of *R. praetextatus* (12.5%) and *R. annulatus* (20.0%). Additionally, an unconfirmed *Babesia* species—the only parasite detected in this study—was identified in *H. rufipes* (4.8%).

**Table 6:** Tick pools positive for additional microorganisms. The microorganisms listed here include *Ehrlichia* spp., *Babesia* sp., *Borrelia* sp., opportunistic *Spiroplasma* spp., *S. ixodetis*, and BPSV. *n* (%) (*A*. = *Amblyomma*, *Hae*. = *Haemaphysalis*, *Hy*. = *Hyalomma*, *Rh*. = *Rhipicephalus*)

	Bacteria					Parasite	Virus
Tick species (*n*)	Opportunistic *Spiroplasma* spp.	*S. ixodetis*	*E. canis*	*E. ruminantium*	*Borrelia* sp.	*Babesia* sp*.*	BPSV
*A. cohaerens* (8)	-	-	-	2 (25.0%)	-	-	-
*Hae. aciculifer* (3)	-	-	-	-	1 (33.3%)	-	-
*Hy. dromedarii* (3)	-	-	1 (33.3%)	-	-	-	-
*Hy. impeltatum* (3)	-	-	2 (66.7%)	-	-	-	-
*Hy. rufipes* (21)	1 (4.8%)	-	-	-	-	1 (4.8%)	-
*Hy. truncatum* (18)	1 (5.6%)	-	-	-	-	-	-
*Rh. annulatus* (5)	-	-	-	-	-	-	1 (20.0%)
*Rh. decoloratus* (13)	4 (30.7%)	1 (7.7%)	-	-	-	-	-
*Rh. evertsi evertsi* (18)	-	-	1 (5.6%)	-	1 (5.6%)	-	-
*Rh. praetextatus* (8)	-	-	-	-	-	-	1 (12.5%)
	6 (3.9%)*	1 (0.6%)*	4 (2.6%)*	2 (1.3%)*	2 (1.3%)*	1 (0.6%)*	2 (1.3%)*

*Overall prevalences were calculated from all tick pools tested (*n* = 154)

## Discussion

Metagenome-based investigations offer the advantage of agnostic detection of any organism found in the sample, unrestricted by the amplification primers without having to target particular genomic regions. Given sufficient copy numbers being present in the sample, multiple pathogens can be identified and discriminated from closely related species. In this first investigation of tick-borne pathogens in Ethiopia, we identified 13 distinct microorganisms among 154 pooled tick samples (Tables 2, 3, 4, 5, and 6). We were also able to specifically identify three opportunistic and endosymbiotic microorganisms (*Coxiella*, *Francisella*, and *Spiroplasma* spp.) that might be challenging to discriminate from closely related pathogens and may incidentally overestimate pathogen prevalence as well as risk of tick-borne disease in the region.

In the case of *Coxiella* spp., there is evidence that the presence of nonpathogenic forms may be interfering with the detection and proper identification of *C. burnetii*, the pathogenic agent causing Q fever*.* This study found higher proportions of *Coxiella-*like endosymbionts, as compared to studies that have targeted select gene regions for identification. In their work on *Coxiella-*like endosymbionts, Körner et al. (2021) highlight the potential for misidentification when screening ticks for pathogenic forms of *Coxiella* using PCR detection methods*,* and Duron (2015) confirms that the popular target, IS1111, is also present and identical in the symbiotic forms. In this study, we found *Coxiella-*like endosymbionts in 53.9% of pooled ticks, while *C. burnetii* was found in only 1.3% of pooled ticks. Previous reports of *C. burnetii* in ticks from Ethiopia using PCR techniques range from 6.4 to 10.8% of tested ticks (Hornok et al. 2014; Kumsa et al. 2015a). Also using PCR, Olivieri et al. (2021) found 28% of ticks collected from Egypt, Ethiopia, and Kenya to be positive for *Coxiella* endosymbionts. Other studies based on targeted short-read sequencing reported prevalences of *C. burnetii* at 45.7% and 37.9% from neighboring Kenya (Ergunay et al. 2022; Kimemia et al. 2024). This serves to reiterate the fact that tick-associated pathogenic bacteria need to be distinguished from their closely related endosymbionts using well-characterized and taxonomically informative regions from the ever-growing and rapidly expanding collection of publicly accessible genome reference databases.

Similar to that observed in Coxiella, pathogenic forms of Francisella may be over-reported in tick-borne pathogen surveillance. Recent work done on *Francisella* spp. highlights the value of genome-wide sequencing in agnostic microorganism discovery and identification. Öhrman et al. (2021) state that of the over 2400 genes that comprise *F. tularensis*, only six are unique across pathogenic and closely related nonpathogenic strains, making targeted approaches to identification difficult. Although 26.0% of all tick pools were positive for *Francisella* species in this study, only 4.5% were found to harbor the pathogenic strain, *F. tularensis novicida*. The remaining positive pools (21.4%) were found with opportunistic *Francisella*, which was more prevalent in *Hyalomma* spp., thus 61.9% (26/42). Using short-read metagenomics, Benyedem et al. (2022) detected nonpathogenic *Francisella* in *Hyalomma* ticks collected from cattle in Tunisia (proportions of positive ticks were not reported). Also using metagenomics, Ergunay et al. (2022) found slightly higher proportions of *F. tularensis* in 14.2% (5/35) of ticks collected from wildlife in Kenya. Other studies have also found nonpathogenic *Francisella* strains in ticks. Using PCR, Olivieri et al. (2021) found 39.4% (28/71) of *Hyalomma* and *Rhipicephalus* ticks collected from wild and domestic animals in Egypt, Ethiopia, and Kenya to be positive for nonpathogenic *Francisella*. In Ethiopia, Szigeti et al. (2014) reported nonpathogenic *Francisella* in one *Hy. rufipes* collected from cattle. The present study not only adds to the record of nonpathogenic and opportunistic *Francisella* being found in ticks collected from Ethiopia, it also provides the first records of the subspecies *F. tularensis novicida* in Ethiopia.

Recent research into tick endosymbionts cautions definitive identification of pathogenic forms of *Coxiella* and *Francisella*. In their work on endosymbionts surveyed from ticks collected worldwide, Duron et al. (2017) found over 20 unique *Coxiella*-like endosymbionts in 30 species across six ixodid tick genera and *Francisella*-like endosymbionts in 13 species across five genera. Our findings demonstrate that the traditionally high levels of *Coxiella* and *Francisella* detection in ticks may be caused by misidentification of closely related nonpathogenic organisms belonging to the same genus as the pathogenic strains. This means that in the broader context of pathogen surveillance, the prevalence of pathogenic agents circulating in the environment and enzootic cycles may be reported at higher levels than what is actually present. This raises perceived risk, which may exhaust surveillance efforts and extend mitigation efforts unnecessarily.

Identifying a particular species within the large group of SFG *Rickettsia* in samples is challenging and requires sequences from multiple genome targets. Aside from *R. africae*, this study could not discriminate between particular species of SFG *Rickettsia* and found 38.3% of tick pools to be positive for one or more of these species, with 14.9% of pools showing evidence for *R. africae.* Using phylogenetic analysis of chromosome and plasmid-located genes, we confirmed two tick pools (*A. gemma* and *A. variegatum*) to be positive for *R. africae* (Fig. 2). Our finding of 38.3% SFG *Rickettsia* among ticks tested is lower than a previous finding of 59.4% *Rickettsia* spp. in ticks from wildlife in Kenya (Kimemia et al. 2024). Although Kimemia et al. (2024) used target-based gene analysis, they did not specify whether the *Rickettsia* found was solely of the spotted-fever group or included typhus-group *Rickettsia*. Our findings of 14.9% *R. africae* among *Amblyomma* ticks tested are in the range of other recent target-gene-based findings in Ethiopia (5.2–28.5% of *Amblyomma* ticks) (Kumsa et al. 2015b; Teshale et al. 2016; Tomassone et al. 2016; Tufa et al. 2021). Another consideration to make when interpreting *R. africae* presence in ticks is the prospect of *R. africae* integration into the genome of *A. variegatum* (Lorusso et al. 2013; Mazhetese et al. 2021). Furthermore, the number of known SFG *Rickettsia* spp. is rapidly expanding, and research has just begun to understand the sheer diversity and respective function of nonpathogenic and symbiotic *Rickettsia* found in ticks (Kipp et al. 2023; Kolo and Raghavan 2023).

*Spiroplasma* spp. were found in 4.5% of pooled samples, with one pool of *Rh. decoloratus* (7.7%) showing positive for the known pathogen *S. ixodetis.* Although Palomar et al. (2022) found *S. ixodetis* in *Rh. decoloratus* (via PCR) collected from Angola and Ergunay et al. (2024) reported *S. ixodetis* in 33.3% (5/15) pools of *Ixodes* ticks (via metagenomics) collected from Mongolia, this is the first known record of *S. ixodetis* in ticks from Ethiopia. Duron et al. (2017) documented six unique *Spiroplasma* spp. to be detected in *Rh. decoloratus*, and although we could not discriminate a particular strain of *Spiroplasma* in our samples aside from *S. ixodetis*, our findings expand the known distribution of *Spiroplasma* spp. found in ticks.

Research by Benyedem et al. (2022) found *Ehrlichia* spp. in ticks from Tunisia using metagenomics, and this is the only known metagenome-based study reporting on *Ehrlichia* spp. from Africa. Our finding of 3.2% *Ehrlichia* spp. among tick pools is lower than 6.9% reported from Kenya (via PCR detection methods) (Omondi et al. 2017).

*Babesia* spp. have been detected in a variety of domestic animals (cattle, dog, donkey goat, and sheep) from many regions of Ethiopia (Amhara, Oromia, South Ethiopia Regional State, and Tigray) (Mekibib et al. 2010; Tefera et al. 2011; Hailemariam et al. 2017; Solomon et al. 2020; Adugna and Tamrat 2022; Fesseha et al. 2022; Tadesse et al. 2023; Tamrat et al. 2023). There are, however, no known records of *Babesia* spp. found in Afar, and the only known detection of *Babesia* (*B. caballi*) in ticks (*A. variegatum*) collected from Ethiopia is documented by Hornok et al. (2014). Our study found one pool of *Hy. rufipes* (4.8%) collected from a camel in the Afar region to be positive for *Babesia* spp. In Egypt, *B. microti* has been detected in camels, but no *Babesia* spp. have been reported in ticks collected from camels in this region (Alsarraf et al. 2017; Ashour et al. 2023). *Babesia* spp. have been detected in *Hyalomma* ticks collected off migratory birds from the Mediterranean region, and the only other account of *Babesia* being detected in *Hy. rufipes* is a report from South Africa (Gray and De Vos 1981; Toma et al. 2014). Some *Babesia* spp. (i.e., *B. occultans*) are considered of low pathogenicity and unlikely to exhibit clinical manifestations and therefore can go undetected (Ros-García et al. 2011; Decaro et al. 2013). Our findings of *Babesia* sp. in an unlikely vector and area should be noted, but additional replication of findings is necessary to determine the extent of distribution for this piroplasm within the high and arid lands of Ethiopia.

This study is the first to document BPSV in ticks collected from Ethiopia, with 1.3% of all pooled ticks and 4.5% of *Rhipicephalus* ticks testing positive. In Burkina Faso, Ouedraogo et al. (2020) found BPSV in 5.8% of tested tick pools, although the study only found *Amblyomma* and *Hyalomma* ticks to have this virus. In France, Cicculli et al. (2022) also found *Parapoxvirus* in 7.9% of tested tick pools, with *Rhipicephalus* being part of the positive pools. BPSV is in the genus *Parapoxvirus*, which currently comprises five unique double-stranded DNA viruses (Cicculli et al. 2022). Viruses belonging to this genus cause lesions on the skin and are spread through direct contact of mucous membranes or open wounds. Although BPSV most likely spreads through direct contact, arthropods may play a role in mechanical transmission, but the vectorial capacity of ticks for this virus is not known (Ouedraogo et al. 2020). Through nontargeted research, we have detected additional tick-borne pathogens that may be unknowingly affecting the health of livestock and should be considered a surveillance target for livestock management.

Although this study showcases the great utility of metagenomic sequencing in diverse, emerging, and potentially novel pathogen detection, it is not without shortfalls. As this is the first known metagenomics study on tick-borne pathogens in Ethiopia, its findings should not be considered a comprehensive assessment of tick-borne pathogens in Ethiopia. This study used samples that were preserved in ethanol at room temperature for nearly a year. Detection of viruses in arthropods can be accomplished with the use of PCR after storage in ethanol; however, the timeline should be limited to a few months (Goffredo and Meiswinkel 2004). For virus detection, researchers should maximize optimal cold-chain storage conditions, beginning with dry-ice cooling of ticks at collection sites (Savage et al. 2013). DNA, as well as RNA, has been shown to degrade faster in ethanol-stored samples as compared to other preservation methods (Fukatsu 1999). Undoubtedly, microbial read quality in this study would have been considerably improved using more appropriate RNA storage solutions, such as RNA later and DNA/RNA Shield.

This study has shown the utility of agnostic metagenomic sequencing in tick-associated microorganism screening when the etiologic agents are unknown. Metagenomic data can be useful when needing to detect a wide breadth of microbial diversity, which yields a broader view of tick-associated microbial fauna. This study has provided additional evidence for tick-microorganism associations, and it shows reliable evidence of microorganism presence can be obtained even with samples that are stored sub-optimally in ethanol at room temperature for extended periods.

The present scarcity in knowledge of free-living and endosymbiotic microorganisms present in arthropods is stark, and agnostic sequencing approaches are increasingly becoming essential tools in the discovery of microorganism diversity across a broad spectrum of hosts. With the widespread availability of sequencing technology, the portability of devices, and the ongoing reductions in sequencing costs per sample, metagenomic approaches now provide a practical and powerful tool for baseline microorganism screening and surveillance of tick-borne disease.

## Supplementary Information

Below is the link to the electronic supplementary material.

Supplementary file1 (XLSX 16 KB)

Supplementary file2 (XLSX 23 KB)

## Data Availability

The following tables are available in the online version of this article: Supplementary File 1: NCBI GenBank® reference genomes used for microorganism identification. Supplementary File 2: Comprehensive list of microorganism findings by pooled sample. Sequences of R. africae used to generate the phylogenetic trees are deposited in the NCBI GenBank® under accession numbers PQ900147, PQ900149, PQ900150, and PQ900151.

## Declarations

### Disclaimer

The material in this article has been reviewed by the Uniformed Services University of the Health Sciences. There is no objection to its presentation and/or publication. The opinions and conclusions contained herein are the views of the authors and do not reflect the views or policies of the Uniformed Services University of the Health Sciences, the Department of the Army, the Department of Defense, or the U.S. Government.
